# The high-throughput production of membrane proteins

**DOI:** 10.1042/ETLS20210196

**Published:** 2021-10-08

**Authors:** James Birch, Andrew Quigley

**Affiliations:** 1 Membrane Protein Laboratory, Diamond Light Source Ltd., Harwell Science and Innovation Campus, Didcot OX11 0DE, U.K.; 2 Research Complex at Harwell (RCaH), Harwell Science and Innovation Campus, Didcot OX11 0FA, U.K.

**Keywords:** high-throughput screening, protein expression, protein purification, structural biology, transmembrane proteins

## Abstract

Membrane proteins, found at the junctions between the outside world and the inner workings of the cell, play important roles in human disease and are used as biosensors. More than half of all therapeutics directly affect membrane protein function while nanopores enable DNA sequencing. The structural and functional characterisation of membrane proteins is therefore crucial. However, low levels of naturally abundant protein and the hydrophobic nature of membrane proteins makes production difficult. To maximise success, high-throughput strategies were developed that rely upon simple screens to identify successful constructs and rapidly exclude those unlikely to work. Parameters that affect production such as expression host, membrane protein origin, expression vector, fusion-tags, encapsulation reagent and solvent composition are screened in parallel. In this way, constructs with divergent requirements can be produced for a variety of structural applications. As structural techniques advance, sample requirements will change. Single-particle cryo-electron microscopy requires less protein than crystallography and as cryo-electron tomography and time-resolved serial crystallography are developed new sample production requirements will evolve. Here we discuss different methods used for the high-throughput production of membrane proteins for structural biology.

## Introduction

**Figure 1.**
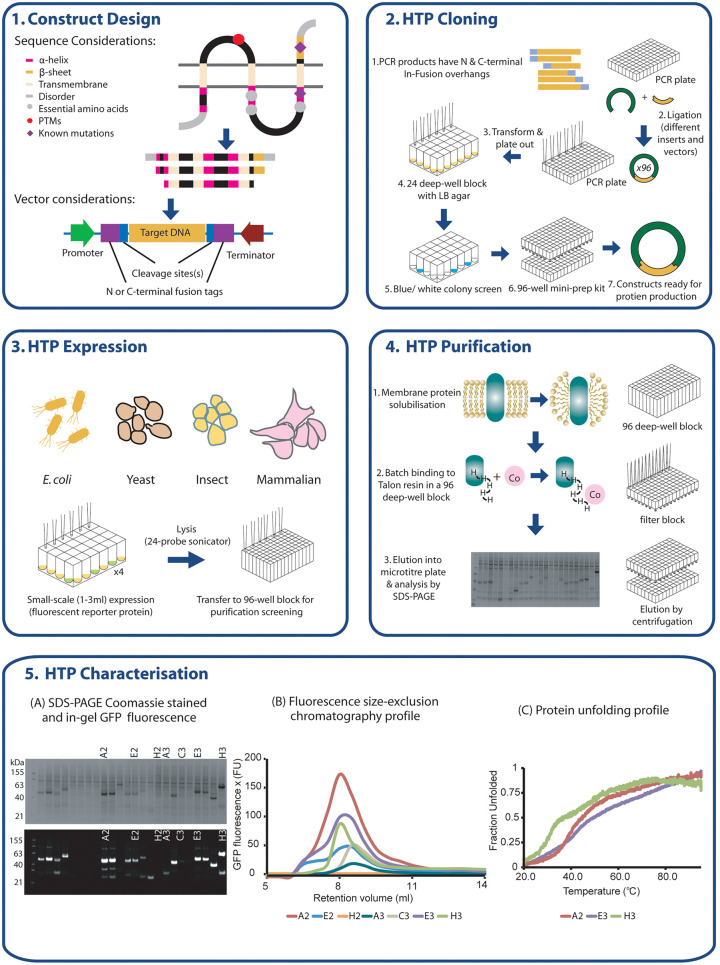
An illustrative overview of the HTP production of membrane proteins. (**Step 1**) Bioinformatics servers and databases such as PSIpred [7] and Uniprot [8] are used to aid construct design, highlighting secondary structural features, domain boundaries critical residues, PTMs and mutant and target isoforms. six–24 constructs are designed for each MP target. Constructs include truncations, (of mainly disordered regions and domain boundaries), functional and disease mutations, and a variety of fusion-tags. (**Step 2**) HTP-cloning enables a diverse library of clones to be established in a few days. (**Step 3**) HTP expression enables the parallel production of all cloned constructs in a few days to 2 weeks depending on expression system. (**Step 4**) HTP purification using 96-well blocks and filter plates to purify 96 different conditions (constructs, encapsulation reagents, solvent conditions or additives) in under 12 h. (**Step 5**) HTP characterisation using (**A**) SDS–PAGE to assess protein purity, yield and susceptibility to proteases. Protein bands can be excised and analysed using mass spectrometry to identify the target protein or contaminants. Here 24 constructs are shown. Strong protein bands are observed for A2, E2 and H3 among others. Protein bands must usually be observable after Coomassie staining as well as GFP fluorescence. For example, although A3 can be detected by GFP florescence, the expected yield would be too low to make this construct tractable. Some degradation is observed for multiple constructs (not uncommon when using a GFP fusion). Note the high sensitivity of GFP. (**B**) F-SEC enables an assessment of monodispersity and provisional oligomeric state. Well-behaved MPs tend to have a clear monodisperse profile but this is not an indication of long-term stability. Seven constructs are labelled here and correspond to bands on the SDS–PAGE gel. GFP is not essential for this analysis. We also use tryptophan fluorescence for MPs lacking GFP tags, especially when GFP removal is destabilising. In these cases twin-strep tags are used to ensure the higher purity required to interpret the F-SEC profiles (**C**) Nano-DSF enables the temperature at which half of a protein sample is folded to be determined and is a good estimate of long-term stability. Ideally, a Tm½ would be more than 40°C. Not all MPs are shown here as low-yielding constructs will not be detected by nano-DSF. A2 and E3 are the most encouraging constructs with good SEC profiles and Tm½ values. Although H3 has a good SEC-profile its thermostability is lower (∼30°C) suggesting that the purification conditions require optimisation.

Constructing a clone that yields sufficient functional protein is the rate-limiting step affecting membrane protein (MP) production necessitating high-throughput (HTP) approaches (Figure 1). Different homologues, truncates and functional mutants should be screened to ensure success. MPs are expressed in prokaryotic and eukaryotic hosts as well as cell-free systems [1–3]. Production yields vary depending on expression system, target protein and production scale but typically between 50 µg and 2 mg of purified protein can be obtained from one litre of cell culture. To place this in the context of structural biology, ∼100–250 µg of protein is required per crystallisation plate whereas 10–20 µg are required for each cryo-electron microscopy (cryo-EM) grid. Many expression systems have been adapted for HTP including full automation [4–6]. The rapid identification of successful constructs and exclusion of those likely to fail is key to an effective approach. HTP MP production has developed significantly over the last two decades and driven the deposition of over 4500 MP structures in the Protein Databank. While HTP methods have addressed some rate-limiting production issues, the process is still largely empirical.

## Manipulating genes for membrane protein expression

Template DNA is the primary prerequisite to generate different expression constructs. Now that the cost of a single base-pair is as little as $0.07 *de novo* DNA synthesis is increasingly used to obtain template DNA. HTP cloning is the only viable way to efficiently generate construct diversity. Although restriction cloning has been adapted for HTP [9], recombination and ligation independent (LIC) approaches are much more reliable. Gateway cloning [10], an example of recombination, enables parallel construction from multiple fragments but causes vector derived amino acid additions. LIC approaches, such as In-Fusion [11] or Gibson assembly [12], utilise large single-strand overhangs in the vector and PCR insert for targeted insertion. The In-Fusion system is our preferred method, enabling one-step, insert-independent, cloning into most expression vectors without process derived amino acid additions. Choice of expression vector is also important [1, 2]. We favour the pOPIN system [13] which supports screening in multiple expression hosts and a wide range of fusion tags (Table 2).

**Table 2:** Fusion-tags that are useful for membrane protein structural and functional studies

Tag	Use	Binding	Advantages	Disadvantages
His-tag	Purification, F-SEC along with NTA linked fluorescent peptides, Purification or experiments that require surface attachment (e.g Surface plasmon resonance)	Nickel/Cobalt/Zinc	Small tag; easy purification	Relatively poor specificity
Fluorescent (GFP, YFP, mCherry)	Tracking MP during expression and purification, purification.	Specific nanobody or antibody	Easy to track protein throughout expression and purification	Large tags; more likely to affect protein function. Antibody/nanobody needed to use for purification
Strep II tag	Purification or experiments that require surface attachment (e.g SPR)	Streptavidin or Streptactin resin	High-affinity and specificity purification	High cost of specific resin
GST tag	Purification or experiments that require surface attachment (e.g SPR)	Glutathione (GSH)	May increase MP yields, useful for pull-downs	Propensity for GST to dimerise
HA	Detection and purification of proteins	HA-specific antibody	Small tag	Tag is cleaved in apoptotic cells
FLAG	Detection and purification of proteins	FLAG-specific antibody	Small tag	High cost of specific resin
ALFA-tag	Detection and purification of proteins	Specific nanobody	Small tag; choice of nanobodies	High cost of specific resin

## Choosing an expression system

Many expression systems successfully produce MPs (Table 1). The most suitable host is usually selected after iterative screening guided by MP target origin. We prefer to express prokaryotic MPs in *Escherichia coli* (*E. coli*) which can be quickly grown in HTP using inexpensive media, in small or large volumes and at high cell density [14]. The bacteriophage T7 promotor, often used for MP expression, can lead to overloading of the bacterial quality control system and accumulation of MPs in inclusion bodies [15, 16]. Mitigation efforts drove the development of alternative bacterial expression hosts [15, 17, 18] and several *E. coli* strains temper the effects of the T7 promoter [19, 20]. Other strains improve the expression levels of specific MPs [21] but MP functionality depends upon the strain [22]. Typically, we screen 4–6 *E. coli* strains to establish the best production conditions.

**Table 1.** Expression systems used to produce membrane proteins, including benefits and drawbacks

Expression system	Benefits	Drawbacks	Comments
*Escherichia coli* (manual induction)	Cheap, well-established technology, minimal equipment needed, effective for many bacterial targets.	Ineffective for most eukaryotic MPs.	Common strains include BL21(DE3), C41(DE3) & C43(DE3) [23], C44(DE3) & C45(DE3) [20] Lemo21(DE3) [19], BL21(DE3) containing either Rosetta2 or Origami.
*Escherichia coli* (auto-induction)	As manual induction except avoids need to monitor OD to add inducer.	Induction occurring after exponential phase can impair expression of some proteins.	Common strains used include many of those listed above. Autoinduction methods described by Studier [24].
*Bacillus subtilis*	Effective for secretion of (non-membrane) proteins into growth medium. Gram positive.	Less well-established than *E. coli*.	One of the original paper describing the use of *B. subtilis* [17].
*Lactococcus lactis*	Improved folding of eukaryotic membrane proteins over *E. coli*	Less well-established than *E. coli*.	Methods for protein production recently described [15, 18].
*Saccharomyces cerevisiae*	Improved expression of eukaryotic MPs.	Expression levels lower than Pichia.	Method that increases MP yield in *S. cerevisiae* [25].
*Pichia pastoris*	Improved folding and PTM of eukaryotic MPs; higher expression levels than *S. cerevisiae*.	Bottleneck due to need to screen many clones. Less suitable for HTP.	Use of *P. pastoris* for MP production [26].
Insect (*Spodoptera frugiperda* & *Trichoplusia ni)*	Improved folding and PTM of mammalian MPs over yeast, yield higher than mammalian cells.	Several weeks needed to generate baculovirus, more expensive than microbial systems. Cell culture lab and expertise needed.	Sf9, Sf21, Hi5, ExpiSf cell lines have been used to express MPs [27, 28].
Mammalian — transient	Ideal for correct folding and PTM of some eukaryotic MPs. Transfection is simple – no need for virus production or cloning/screening.	More expensive than microbial systems. Cell culture lab and expertise needed. Low yields. Large amounts of transfection-grade plasmid DNA needed for scale-ups.	Recent protocol production eukaryotic MP in Human embryonic kidney (HEK) cells [29]. An automated transient approach [5].
Mammalian — BacMam	Ideal for correct folding and PTM of some eukaryotic MPs. Useful for large-scale production.	More expensive than microbial systems. Cell culture lab and expertise needed. Low yields. Virus production time consuming.	Recent protocol describing use of the BacMam system for MP production [30].
Mammalian — stable	Ideal for correct folding and PTM of some eukaryotic MPs. Avoids requirement for large amounts of DNA or virus.	More expensive than microbial systems. Cell culture lab/ expertise needed. Low yields. Slower than transient. Lentiviral systems require containment at early stages.	Recent lentiviral protocol [31]. An automated stable approach [5].
Cell-free	Expression of highly toxic proteins possible. MP directly incorporated into encapsulation agents.	Cost prohibitive if large amounts needed.	Cell-free systems have been adapted from yeast, wheatgerm, insect and mammalian expression hosts [3, 32]. Also adapted for automation [6].

Eukaryotic MPs are expressed in prokaryotic and eukaryotic hosts [2]. We generally avoid prokaryotic hosts as they lack essential lipids and machinery for post-translational modifications (PTMs). Other microbial expression systems including *Saccharomyces cerevisiae* [25] and *Pichia pastoris* [26] have been employed when bacterial systems fail. Yeast are cheaper, easier to genetically manipulate and grow than other eukaryotic hosts, are capable of some essential PTMs [2], but lack essential lipids, impairing human MP production [33]. Insect cells present an alternative to yeast [27, 28] but use baculoviral vectors for construct delivery which is time consuming [28]. We find that the ExpiSf system [34] improves production efficiency by maintaining higher cell densities. However, insect cells have a less well-regulated quality control system that results in accumulation of unfolded, inactive protein compared with mammalian cell lines [35]. Transient transfection, baculovirus transduction or stable transfection facilitate MP expression in mammalian hosts [5, 30, 36]. During initial screening, we use transient transfection, which utilises chemical agents to introduce non-integrating plasmid DNA into cells. Coupled with reporter proteins, such as green fluorescent protein (GFP), expression parameters can be screened rapidly [29]. For large-scale production, baculoviral and stable cell systems are more cost effective. Traditionally stable cell lines were unsuitable for HTP expression as the generation of monoclonal cell lines took up to six months. Recently [31, 36], a lentivirus was adapted to enable inducible MP expression in polyclonal stable cell lines, achieving a transfection efficiency approaching 100%. Polyclonal stable cell lines are generated on a similar timeframe to baculoviral methods. Along with other rapid stable approaches [5, 37] the lentivirus system presents an exciting opportunity for the future HTP MP production.

## Establishing a purification method

HTP purification methods tend to be restricted to small-scale screens that are used to identify the most suitable purification conditions prior to scale-up. Purifying a MP is in essence no different from any other protein. Common purification tags, proteases for tag removal and affinity resins are used for membrane and soluble proteins. Extraction of the MP from the host membrane is the exception, with detergents commonly used for this purpose. Selecting the most suitable detergent is not always compatible with the ideal encapsulation reagent required for biological analysis, necessitating exchange. Detergents with high critical micelle concentrations (CMCs) are easily exchanged by dialysis whereas hydrophobic beads are used at low CMCs. Maltosides such as dodecyl-maltoside (DDM) enable efficient extraction and yield functional protein making the maltoside class some of the most commonly used detergents in structural biology [38]. Hundreds more detergents have been developed to stabilise MPs further leading to HTP screens to identify the most useful [39, 40]. Recently introduced detergents include the neopentyl glycols [41] and glyco-diosgenin (GDN) which along with digitonin account for around a third of single-particle cryo-EM (cryo-SPA) structures. The recently developed oligoglycerol class is useful for native mass spectrometry [42]. Although detergents are commonly used to manipulate MPs, drawbacks including disruption of tertiary and quaternary structure, MP inactivation and occlusion of purification tags has led to the development of other encapsulation reagents. These reagents include amphipols [43], membrane scaffold proteins (MSPs) [44], co-polymer lipid particles [45, 46], Saposin A [47], peptidiscs [48] and proteoliposomes. MSPs and amphipols are particularly effective agents for cryo-EM and useful encapsulation reagents for nuclear magnetic resonance spectroscopy [49] and small-angle X-ray and neutron scattering [50]. Co-polymer lipid particles can directly solubilise MPs without detergents, retaining native lipids, and have been used for cryo-SPA [51], lipidic cubic phase (LCP) crystallisation [52], functional assays [51] and biophysical characterisation [53]. Lipids such as Cholesteryl hemisuccinate are commonly used to stabilise MPs [38].

Fusion-tags for protein production range from a few amino acids e.g. polyhisitdine tags (his-tags) to small proteins such as GFP (Table 2). His-tags enable affinity purification and comprise six to ten histidine residues. Background contamination can be high when using his-tags [54] and we recommend a reverse purification step. For one-step purification we prefer twin-strep-tags [55] which are ideal for HTP screening platforms [30]. GFP-specific nanobodies and megabodies also enable one-step purification for GFP tagged MPs [56, 57]. All fusion tags can interfere with MP function, propensity to crystallise and may affect yield. Fusion tags such as lysozyme aid GPCR crystallisation [58]; however, fusion tag removal can be an important step in eliminating contaminants through a reverse purification step. Many proteases (Table 3) used to cleave MP fusion tags have reduced efficiency in the presence of some detergents [59, 60]. We favour the Tobacco etch virus (TEV) and Human rhinovirus (HRV) 3C proteases as both have stringent sequence specificity, are functional at 4°C and are easily producible at scale in the laboratory. The Tag-on-demand system utilises amber-codon suppression to enable tags to be ‘turned on and off’ when expressed which is advantageous for many drug screening platforms [61].

**Table 3:** Common proteases used for fusion tag removal

Protease	Advantages	Disadvantages
TEV	Stringent cleavage-sequence specificity and few residual amino acids after cleave. Easily produced in house. Good activity in a range of buffers and at 4°C.	Activity limited by some commonly used detergents. Reducing agents required for activity. Comparatively low activity.
HRV 3C	Stringent cleavage-sequence specificity and few residual amino acids after cleave. Easily produced in house. Good activity in a range of buffers and at 4°C.	Activity limited by some commonly used detergents. Comparatively low activity.
Thrombin	Not affected by the majority of detergents.	Non-specific cleavage, Inhibited by reducing agents and common protease inhibitors used during purification.
SUMO protease	No recombinant linker region needs to be constructed, native N-terminus of the target protein is maintained.	Little activity in many commonly used detergents.
Factor Xa	Not affected by the majority of detergents.	Non-specific cleavage, Inhibited by reducing and chelating agents, phosphate ions.

## Assessing the quality of expressed membrane proteins

Protein engineering has been used to produce highly stable MP constructs [62, 63]. The alternative is the use of HTP screens to identify the most stable conditions for purification. Either way the tag-dependant assessment of MP quality and quantity in cells, solubilised lysates or using purified protein is essential. Expression alone is a good indicator of failure but a poor indicator of success, as a MP's stability or functional state is not considered. We use a simple pulldown approach, after solubilisation, to obtain pure protein using 96-well blocks to screen many conditions such as constructs, encapsulation agents, solvents and lipids in parallel. Purified MPs are analysed by sodium dodecyl sulfate polyacrylamide gel electrophoresis (SDS–PAGE), fluorescence size exclusion chromatography (F-SEC), differential scanning fluorimetry [40] and mass spectrometry [64]. These techniques can collectively assess yield, monodispersity, thermostability, PTMs and, identify the target. Multichannel pipettes (or robotics) and careful plate layouts help avoid cross-contamination while mass spectrometry is used to flag any cross-contamination events. To track MPs during expression and purification we use C-terminal GFP fusions [65, 66]. Whole-cell GFP fluorescence correlates well with overall protein yield [67]. GFPtags also give a crude indication of protein folding and localisation, and enable assessment of monodisperisty and stability, by F-SEC, in different encapsulation reagents. However, GFP tags cause both false positives and negatives as GFP is highly stable and can reduce expression levels [54]. A recently developed multivalent nitrilotriacetic acid fluorescent probe [68] enables GFP-free screening but background histidine rich proteins can interfere. Alternatively, a fluorescently tagged nanobody raised against the ALFA tag has enabled GFP-free screening following a one-step purification method [69].

## Changing requirements for membrane protein production

Future iterative developments to MP production will improve efficiency, reduce costs and boost yields. Currently beyond the reach of many labs, cell-free expression systems [6, 70] present a different approach that enables the rapid production of functional MPs directly incorporated into nanodiscs or proteoliposomes without need for comprehensive production strategies. Nanodiscs have already proven their utility for cryo-SPA and proteoliposomes were recently used when solving the structure of AcrB [71]. New purification strategies could speed up the purification of MP samples [72].

New challenges will be placed on MP production as structural approaches are developed. HTP production strategies are ideally placed to address these challenges through the parallel production of multiple constructs for different applications. New techniques may also bring advantages; the (cryo-SPA) resolution revolution has enabled new MP structures and requires less sample than crystallography. Nanobodies can facilitate cryo-SPA of smaller MPs [73]. New nano-focus beamlines for X-ray crystallography and micro-electron diffraction will allow structures to be solved from smaller MP crystals, at higher resolutions [74]. Electron cryo-tomography will facilitate atomic resolution structures, solved directly from cells and tissue, negating the need for solubilised and purified MPs [75]. Alone, each structural methodology is an effective tool to interrogate structure and function. Together, through the power of integrative structural biology, disparate datasets will be brought together to produce improved dynamic and spatial models supporting biomedical discoveries. Thus, the production of high-quality MPs will continue to be essential.

## Abbreviations

CMCs: critical micelle concentrations
F-SEC: fluorescence size exclusion chromatography
GFP: green fluorescent protein
HRV: Human Rhinovirus
HTP: high-throughput
MP: membrane protein
MSPs: membrane scaffold proteins
PTMs: post-translational modifications
TEV: Tobacco Etch virus
